# Foreign Correspondence

**Published:** 1871-12

**Authors:** 


### FOREIGN CORRESPONDENCE.

In a private letter from our friend, Dr. A. W. Calhoun, of Newnan, Georgia, now in Berlin, Prussia, we find the following interesting professional item:

"Berlin escaped the cholera, which scourged some portions of Europe, and especially so of Germany. But she is now suffering from an epidemic of small-pox, which is more violent and fatal than has occurred in a great number of years. Instead of succumbing to the present mode of treatment, it daily grows worse, and threatens to equal the cholera in its ravages. An usually large per cent. of the cases die, and one of the physicians at the "Small-Pox Hospital" informs me that the rate of mortality increases at a fearful rate.

"All the schools are under good headway, and we are kept busy from morning until night in attendance upon clinics and lectures. From all accounts, the number of students exceeds that of any previous year in the history of Berlin. Americans are in the greatest abundance—so much so, in fact, that one almost imagines himself for a time in America; and what impresses one immediately is the most marked respect paid to them, individually and collectively, *as Americans*."

We are specially surprisəd at the facts stated in regard to the occurrence and fatality of small-pox in a city where it has been supposed that vaccination was systematically and thoroughly *enforced*.

Dr. Calhoun promises us some correspondence for the pages of the JOURNAL, which, we have no doubt, will be both interesting and instructive. We have, also, reason to expect some interesting correspondence from Dr. Isaac Ott, of Easton, Pennsylvania, who has recently left for Germany, where he expects to spend some time, and will then proceed to Paris, and perhaps to other important medical centers of Europe. Dr. Ott is a corresponding member of the Atlanta Academy of Medicine, and has contributed several articles to one or more medical journals, and is regarded as a good writer.

